# Advancing Perioperative Medicine With AI: Current Landscape and Research Priorities

**DOI:** 10.2196/107155

**Published:** 2026-10-30

**Authors:** Nidhi Rohatgi, Piyush Mathur

**Affiliations:** 1 Department of Medicine, Stanford University, 300 Pasteur Drive, Palo Alto, CA, 94304, United States, 1 6507234000; 2 Department of Anesthesiology, Cleveland Clinic, Cleveland Clinic Lerner College of Medicine, Cleveland, OH, United States

**Keywords:** artificial intelligence, perioperative medicine, risk prediction, machine learning, clinical decision support

## Abstract

AI is increasingly being applied across the perioperative continuum. AI should be viewed as a technology that can reduce cognitive burden, collate fragmented data into clinically meaningful information, support decision-making, and extend precision education to clinicians and patients. This editorial accompanies the *JMIR Perioperative Medicine* webinar held in May 2026 and presents our perspectives—informed by our clinical experience—on where AI can assist perioperative clinicians and surgical patients, the evidence available today, what is still needed, and how clinicians can prepare to practice alongside AI tools. We consider opportunities in preoperative risk assessment and optimization; intraoperative intelligent decision support; emerging technologies, such as surgical digital twins; and postoperative recovery, monitoring, and prediction of clinical deterioration. Most AI applications remain early and require large-scale, prospective validation across diverse populations and resource settings. Realizing their potential also requires confronting substantial challenges, including limited and fragmented training data, the specialized models needed to interpret the large volumes of data captured in operative videos and during physiological monitoring, biases embedded in data and algorithms or biases inherent in human-AI interactions, and the determination of where human oversight is essential and where it is not feasible. When used responsibly, with human judgment remaining paramount, AI offers an unprecedented opportunity to advance perioperative care.

## Introduction

AI is becoming a defining technology in health care. AI can reduce cognitive burden, improve efficiency, enhance learning, and enable clinicians to devote time and attention to the uniquely human aspects of care and the contexts where human judgment is indispensable.

The perioperative continuum begins days or months prior to surgery and extends through the intraoperative course, recovery, and rehabilitation, with focus shifting to follow-up beyond the traditional 30 or 90 days after surgery. AI is evolving from a collection of task-specific algorithms to a longitudinal intelligence system that can support care delivery and decision-making across the perioperative continuum. This evolution was reflected throughout the *JMIR Perioperative Medicine* webinar, “Advancing Perioperative Care with Artificial Intelligence,” held in May 2026*,* which emphasized a practical perspective grounded in the real-world implementation of AI [1]. This accompanying editorial—written for perioperative researchers and clinicians—discusses our perspectives on where AI can assist or augment perioperative clinicians and surgical patients, what is the evidence we have, what is still needed, and how perioperative clinicians can prepare to practice with AI. Across the preoperative, intraoperative, and postoperative phases, the recurring messages are that the nearest-term value of AI lies in synthesizing fragmented data and not in autonomous decision-making, most applications await prospective validation in diverse real-world settings, and human judgment remains paramount.

## Preoperative Opportunities

Contemporary preoperative risk assessment involves history-taking focused on multiorgan system assessment, the review of records within and outside the electronic health record (EHR), outreach to the patient’s ambulatory clinicians and caregivers, medication management, and appropriate consultations. These data are then integrated with the surgical complexity; the indication for surgery; the patient’s goals, expectations, and functional, nutritional, psychosocial, and cognitive status; preoperative risk indices; pharmacogenomic data; current evidence; and the clinician’s judgment, to formulate individualized plans for preoperative optimization and intra- and postoperative management. When conducted thoroughly and in partnership with the patient, their caregivers, and the clinicians involved in their longitudinal care, preoperative evaluation offers an unmatched opportunity to prevent postoperative complications [2]. However, it is time-consuming and requires nuanced expertise.

## Information Synthesis From the EHR

AI’s key value in preoperative assessment is in collating data across multiple fragmented data streams and synthesizing them into meaningful information [3]. AI can retrieve relevant clinical practice guidelines to suggest targeted tests and interventions and compute risk scores. Conversational AI agents are an emerging opportunity for history-taking, although in one study, patients provided fewer details to AI agents than to humans [4]. Ambient intelligence has reduced the cognitive burden during documentation, although time savings, the quality of AI-scribed notes, and adoption vary considerably [5-9]. In a randomized crossover trial, the Perioperative AI Chatbot (PEACH) did not reduce the overall documentation time for resident physicians, but time savings were noted for moderate-complexity patients and for more experienced physicians [9]. With AI, clinicians’ effort can shift from information retrieval, documentation, and administrative tasks toward shared decision-making and reasoning.

## Multimodal Risk Prediction

Importantly, only a fraction of a patient’s trajectory is captured in the EHR. Most physiological variation occurs outside clinics and hospitals. Often, patients themselves possess the richest source of health information. Wearables, patient-reported outcomes, and mobile health apps can incorporate data generated during everyday life rather than data generated exclusively during episodes of illness [10]. This transition from episodic “sick care” toward continuous health monitoring may reshape preoperative optimization and long-term outcomes.

AI applied to wearable accelerometry data may be able to predict frailty, which is central to preoperative risk stratification [11]. The AI-assisted identification of sarcopenia from routine computed tomography and AI-assisted coronary calcium estimates could also enhance preoperative risk assessment [12-14]. Beyond the perioperative episode, AI-assisted retinal scans can predict the long-term risk of major adverse cardiovascular events [15]. In a primary care setting, an AI-enabled stethoscope improved the detection of heart failure, valvular heart disease, and atrial fibrillation (all are important perioperative diagnoses), although adoption declined over time without workflow integration [16]. Further, AI-guided echocardiograms are showing promise in improving access to patients by improving the efficiency and effectiveness of trained sonographers and providing real-time guidance to novice sonographers [17,18].

Electrocardiograms (ECGs) are often obtained preoperatively. In a retrospective cohort, deep learning applied to preoperative ECGs discriminated postoperative mortality with an area under the curve (AUC) of 0.83, whereas the AUC for the Revised Cardiac Risk Index was 0.67 [19]. In the nonsurgical setting, the self-supervised interpretation of ECGs across 77 cardiac conditions achieved AUCs of 0.98‐0.99 [20]. Another AI-ECG model achieved AUCs of 0.90‐0.91 for detecting right ventricular and low left ventricular systolic dysfunction [21]. AI-interpreted ECGs and AI-assisted focused ultrasound could potentially reduce unnecessary preoperative echocardiograms [22]. Whether acting on AI-interpreted ECG findings improves clinically meaningful outcomes or cascades low-yield evaluation (eg, echocardiograms and cardiology consultations) requires prospective validation across institutions with varying disease prevalence and surgical risk profiles [23].

## Evaluation of AI-Based Risk Predictions

Predictive analytics is a common application of AI in perioperative medicine, although a systematic review of 103 predictive analytics machine learning (ML) tools reported that 75% of them were validated at a single center, only 13% were externally validated, and the PROBAST (Prediction Model Risk of Bias Assessment Tool) assessment showed a high risk of bias [24]. Preoperative risk indices have largely utilized structured data and regression techniques, including the American College of Surgeons’ surgical risk calculator, which was later enhanced by using ML [25]. The performance of these risk indices has been variable and often moderate across different surgeries and populations. Some institutions have developed their own perioperative risk algorithms by using ML, such as MySurgeryRisk, which predicts 8 major postoperative complications and mortality by using automated EHR input [26,27]. That said, risk prediction alone can be insufficient. In the Perioperative Outcome Risk Assessment with Computer Learning Enhancement (Perioperative ORACLE) randomized trial, access to perioperative ML prediction models did not improve clinician performance, as clinicians anchored to their own predictions [28]. Additionally, effective perioperative care requires actionable pathways and infrastructure for the optimization of those at risk.

## Intraoperative Opportunities

AI is being explored to predict surgical case duration and cancellation and forecast surgical volumes for staffing [29]. Intraoperative care generates enormous volumes of data from hemodynamic monitoring, neuromonitoring, ventilators, closed-loop drug delivery systems, and operating room black boxes implemented at some institutions. Yet, capturing and extracting these data in a form usable for training AI can be challenging or expensive, or they can be beyond the context limits of general-purpose foundation models.

The operating room is a high-risk environment, and AI remains in the early phases of exploration. In the operating room, human-in-the-loop is particularly challenging when precious minutes must be spent on intervention rather than on assessing the accuracy, assumptions, or confidence of AI output. The interface of AI output therefore deserves special consideration, alongside sharing with the surgeon or anesthesiologist what data and evidence were used to train the models.

## Intraoperative Risk Prediction

For anesthesiologists, real-time, AI-driven decision support and monitoring can reduce intraoperative cognitive burden [30]. The Hypotension Prediction Index—an ML algorithm applied to arterial waveform analysis—can warn about impending hypotension a few minutes before the event, allowing for preemptive intervention, but the benefit for patient outcomes is yet to be established [31].

## Intraoperative Intelligence and Precision Education

AI is emerging to assist with medication safety checks in the operating room, provide ultrasound guidance during regional blocks, and anticipate transfusion needs [32-34]. Surgery is being reshaped by robotics and AI-augmented tumor detection [35-38]. Computer vision can analyze operative videos to teach technical skills, deliver feedback, and improve ergonomics [39-41]. Further, specialized surgical video language models can review hours of footage, extending precision education and performance insights beyond what the manual review of surgical videos ever allowed [42].

## Simulation With Digital Twins

Another emerging frontier is the surgical digital twin that can be a virtual replica of the patient, their physiology, or the operating room itself [43,44]. Such digital twins are being explored for surgical planning, training, and real-time guidance with patient-specific anatomy. With digital twins, in silico trials can optimize processes and surface failure modes in a virtual, risk-free, and cost-effective environment. The central challenge is data that may be protected (eg, EHR and imaging data) or data that are never captured because of a lack of sensors, haptics, optical tracking, videos, or extended reality. These models also demand significant computational power.

## Postoperative Opportunities

Preoperative assessment remains critical for anticipating, preventing, and managing postoperative complications. The risk of short- and longer-term adverse postoperative outcomes concentrates among patients with the highest burden of chronic conditions or geriatric syndromes [45,46]. Among older adults undergoing major elective noncardiac surgery, 1 in 5 patients may sustain significant new disability or die by 1 year after surgery [45].

## AI-Based Postoperative Monitoring

Several ML-based early warning systems have been prospectively validated and integrated into clinical workflows [47,48]. For example, the Targeted Real-Time Early Warning System (TREWS) system for the prediction of sepsis across 590,000 medical and surgical inpatients was associated with a 5.1% adjusted absolute reduction in the in-hospital mortality rate if TREWS alerts were confirmed within 3 hours by a clinician [47]. Extending these models specifically to postoperative patients, in whom altered physiology and certain laboratory abnormalities are a normal and expected part of recovery, is an opportunity. Tachycardia, hypoxia, anemia, and leukocytosis carry a different differential diagnosis for a postoperative patient compared to that for a medical patient, and they often reflect a combination of surgery-specific and medical factors. ML-based prediction models have also been developed to predict postoperative acute kidney injury [49].

Because postoperative patients carry particular risks from their underlying disease, the procedure, and the combination of opioids and residual anesthesia, intermittent (and sometimes inaccurate) vital sign measurements in general wards are especially likely to miss deterioration. Continuous monitoring detects far more postoperative deterioration than intermittent checks, including opioid-induced respiratory depression, hypotension, and hypoxemia, and deep learning could be applied to these continuous monitoring−provided data [50,51]. Whether earlier detection reduces complications, unplanned intensive care utilization, or death is unestablished.

## Informed Patients

AI can help educate patients in their preferred language, format, and health literacy level, potentially reducing surgical cancellations and improving patient experiences and outcomes. After surgery, recovering patients must navigate complicated instructions, manage multiple conditions, follow up with several specialties, and sometimes recover at rehabilitation facilities. AI can improve discharge education and extract meaningful information from remote monitoring. AI is being explored to generate discharge summaries and draft responses to patient messages [52-54]. Additionally, conversational AI agents are being explored to provide discharge education. In a randomized trial of 261 orthopedic surgery patients, a context-specific AI agent deployed through a mobile app showed promise in improving postoperative functional recovery and patient experiences in the first few months after orthopedic surgery, when compared with physician-only communication [55].

## Postdischarge Management

More than half of 30-day postoperative complications occur after discharge, which is when patients are least monitored and have the least resources [56]. The postoperative phase requires functional, nutritional, and cognitive rehabilitation; wound management; and pain management. AI is being explored to detect surgical site infections in patient-submitted images [57]. By incorporating the patient’s unique medical, psychosocial, and genomic profile, alongside patient-reported feedback, AI could potentially identify those at high risk of uncontrolled postoperative pain or persistent opioid use and personalize opioid tapering and nonopioid analgesic management plans [58-62].

AI is showing promise in predicting readmission and acute care utilization by incorporating unstructured EHR data and social determinants of health, going beyond traditional structured data [63-65]. However, challenges with data fragmentation resulting from a lack of EHR interoperability across the different hospitals, clinics, and rehabilitation facilities where surgical patients may receive care, or those resulting from a lack of digitized records, remain unsolved.

## Conclusion

AI presents an unprecedented opportunity to advance perioperative care. Most perioperative AI applications remain in the early phases of research and development. Rigorous, large-scale, prospective validation in real-world environments and across diverse populations, geographic regions, and resource settings is still needed. Despite the promise of AI in augmenting perioperative care, human vigilance, situational awareness, and judgment remain paramount. Perioperative clinicians should engage with AI; understand its fundamentals; use it as a thought partner; and, when feasible, engage in the evaluation and validation of AI. Clinicians should continue to hone their clinical skills, as discordance between clinical judgment and a confident AI output will demand even greater expertise. As AI assumes more cognitive and procedural tasks, the loss of some skills is inevitable, as is the acquisition of new competencies. The priorities are to maintain proficiency in the skills critical for patient safety and to remain capable of practicing effectively across settings with varying levels of AI adoption.

## Abbreviations

AUC: area under the curve
ECG: electrocardiogram
EHR: electronic health record
ML: machine learning
PEACH: Perioperative AI Chatbot
Perioperative ORACLE: Perioperative Outcome Risk Assessment with Computer Learning Enhancement
PROBAST: Prediction Model Risk of Bias Assessment Tool
TREWS: Targeted Real-Time Early Warning System
